# Comparison of deregulated expression of cyclin D1 and cyclin E with that of cyclin-dependent kinase 4 (CDK4) and CDK2 in human oesophageal squamous cell carcinoma

**DOI:** 10.1038/sj.bjc.6690348

**Published:** 1999-04

**Authors:** M Matsumoto, M Furihata, T Ishikawa, Y Ohtsuki, S Ogoshi

**Affiliations:** Departments of Pathology II, andSurgery II, Kochi Medical School, Nankoku, Kochi 783-8505, Japan

**Keywords:** cyclin, cyclin-dependent kinase, oesophageal squamous cell carcinoma

## Abstract

The expressions of cyclin D1, cyclin E, cyclin-dependent kinase 4 (CDK4), and CDK2 were immunohistochemically examined in 90 patients with human oesophageal squamous cell carcinoma (SCC) to determine their relationship to the tumour behaviour and patient prognosis. Nuclear immunostaining of cyclin D1 and cyclin E was observed in 28 (31.1%) and 27 tumours (30.0%) respectively. Thirty-nine tumours (43.3%) and 31 tumours (34.4%) exhibited both cytoplasmic and nuclear positivity for CDK4 and CDK2 respectively. Of 28 cyclin D1-positive and 27 cyclin E-positive tumours, CDK4 was overexpressed in 12 (42.8%) tumours and CDK2 in seven (25.9%) tumours respectively. There was no significant relationship in immunopositivity between cyclin D1 and CDK4 or between cyclin E and CDK2. Simultaneous immunoreactivity for both cyclin D1 and CDK4 was significantly associated with venous invasion (*P* < 0.05). In a univariate analysis, the prognosis of patients with tumours that were both cyclin D1- and CDK4-positive was significantly poorer than that of patients with cyclin D1-negative tumours (*P* < 0.05). In a multivariate analysis, both cyclin D1 and CDK4 immunoreactivities (*P* < 0.01) and tumour stage (*P* < 0.001) were recognized as independent risk factors. In this analysis, the hazard ratio for cyclin D1-positive and CDK4-negative cases compared with cyclin D1-negative cases was significant (hazard ratio = 3.128, 95% confidence interval = 1.418–6.899, *P* = 0.0047). No significant prognostic relevance was detected in both cyclin E and CDK2 immunoreactivity. Our in vivo findings suggest that in human oesophageal SCC, cyclin D1 and cyclin E and their functional partners, CDK4 and CDK2, often exhibit dysregulated overexpression in many cases, and that tumours with simultaneous expression of cyclin D1 and CDK4 are frequently associated with venous invasion and have a worse prognosis, statistically. Moreover, overexpression of cyclin D1 alone may also contribute to tumour progression independent of CDK4 overexpression. © 1999 Cancer Research Campaign


*British Journal of Cancer* (1999) **80**(1/2), 256–261
© 1999 Cancer Research Campaign
Article no. bjoc.1998.0348

### Comparison of deregulated expression of cyclin D1 and cyclin E with that of cyclin-dependent kinase 4 (CDK4) and CDK2 in human oesophageal squamous cell carcinoma

**M Matsumoto[1,2], M Furihata[1], T Ishikawa[2], Y Ohtsuki[1] and S Ogoshi[2]**

Departments of [1]Pathology II and [2]Surgery II, Kochi Medical School, Nankoku, Kochi 783-8505, Japan

**Summary** The expressions of cyclin D1, cyclin E, cyclin-dependent kinase 4 (CDK4), and CDK2 were immunohistochemically examined in 90 patients with human oesophageal squamous cell carcinoma (SCC) to determine their relationship to the tumour behaviour and patient prognosis. Nuclear immunostaining of cyclin D1 and cyclin E was observed in 28 (31.1%) and 27 tumours (30.0%) respectively. Thirty-nine tumours (43.3%) and 31 tumours (34.4%) exhibited both cytoplasmic and nuclear positivity for CDK4 and CDK2 respectively. Of 28 cyclin D1-positive and 27 cyclin E-positive tumours, CDK4 was overexpressed in 12 (42.8%) tumours and CDK2 in seven (25.9%) tumours respectively. There was no significant relationship in immunopositivity between cyclin D1 and CDK4 or between cyclin E and CDK2. Simultaneous immunoreactivity for both cyclin D1 and CDK4 was significantly associated with venous invasion ($P < 0.05$). In a univariate analysis, the prognosis of patients with tumours that were both cyclin D1- and CDK4-positive was significantly poorer than that of patients with cyclin D1-negative tumours ($P < 0.05$). In a multivariate analysis, both cyclin D1 and CDK4 immunoreactivities ($P < 0.01$) and tumour stage ($P < 0.001$) were recognized as independent risk factors. In this analysis, the hazard ratio for cyclin D1-positive and CDK4-negative cases compared with cyclin D1-negative cases was significant (hazard ratio = 3.128, 95% confidence interval = 1.418–6.899, $P = 0.0047$). No significant prognostic relevance was detected in both cyclin E and CDK2 immunoreactivity. Our in vivo findings suggest that in human oesophageal SCC, cyclin D1 and cyclin E and their functional partners, CDK4 and CDK2, often exhibit dysregulated overexpression in many cases, and that tumours with simultaneous expression of cyclin D1 and CDK4 are frequently associated with venous invasion and have a worse prognosis, statistically. Moreover, overexpression of cyclin D1 alone may also contribute to tumour progression independent of CDK4 overexpression.

**Keywords:** cyclin; cyclin-dependent kinase; oesophageal squamous cell carcinoma

Molecular biological studies have recently revealed that various molecules including cyclins, cyclin-dependent kinases (CDKs) and CDK inhibitors (CDKIs) play important roles in controlling major checkpoints in the mammalian cell cycle. At least nine classes of cyclins and seven CDKs, including G1 cyclins (D1–3, E and A) and their catalytic partners, CDKs (2, 4 and 6), have now been isolated (Sherr, 1993, 1994; Nakamura et al, 1995; Weinberg et al, 1995). CDK4 and CDK6 are activated by formation of a complex with D-type cyclins which acts as a growth sensor, phosphorylates, and inactivates pRB, the product of the retinoblastoma tumour suppressor gene (Sherr, 1994). Unphosphorylated pRB binds to and inactivates transcription factors including E2F, and prevents the G1–S transition, whereas phosphorylated pRB prevents the interaction of pRB with E2F and enables it to promote gene expression (Weinberg et al, 1995). CDK2 binds to cyclin E or cyclin A, and also inactivates pRB and regulates G1–S phase (Sherr, 1994; Weinberg et al, 1995). On the other hand, CDKs are negatively regulated by the CIP/KIP family of CDKIs including p21, p27 and p57, and by INK4 proteins including p15, p16, p18 and p19 (Sherr, 1996). The CIP/KIP family combines with the cyclin–CDK complex and inactivates it, whereas INK4 proteins directly form complexes with CDK4 and CDK6 and inactivate them and regulate G1 progression (Sherr, 1994, 1996; Lee et al, 1995; Weinberg et al, 1995).

Recently, alteration of the genes encoding these cell cycle regulators have, along with oncogenes and tumour suppressor genes, been reported to contribute to oncogenesis (Sherr, 1996). In various human tumours, aberrant expressions of these cyclins, CDKs, and CDKIs have been reported, and it has been suggested that the loss of cell cycle regulation due to dysregulated expression of these proteins, especially those in the CDK4/cyclin D1-pRB pathway, directly contributes to tumorigenesis (Motokura et al, 1993; Bartkova et al, 1996; Sherr, 1996).

Previous studies of human oesophageal squamous cell carcinoma (SCC) have presented evidence of overexpression of cyclin D1 at the DNA, mRNA or protein level in addition to altered expression of pRB, p53 (Jiang et al, 1993; Tsuruta et al, 1993; Igaki et al, 1994; Wang et al, 1994; Naitoh et al, 1995). It has been also reported that there was a good relationship between cyclin D1 protein expression and gene amplification (Sheyn et al, 1997). Our findings have also demonstrated a relationship between the expression of cyclins, especially cyclin D1 and cyclin E, and various clinicopathological factors relevant to patient prognosis (Furihata et al, 1996; Ishikawa et al, 1998). In addition, both of the tumour suppressor genes, p53 and pRB, appeared to be involved in

*Received 3 June 1998*
*Revised 13 October 1998*
*Accepted 5 November 1998*

*Correspondence to:* M Furihata tumorigenesis (Furihata et al, 1993; Ishikawa et al, 1997). Although CDK4, cyclin E and CDK2 are thought to play important roles in tumorigenesis (Khatib et al, 1993; Marone et al, 1998), no detailed studies have been performed including the immunohistochemical detection and prognostic relevance of these proteins in human oesophageal SCC.

In this study, we immunohistochemically examined 90 cases of human oesophageal SCC to elucidate the immunoreactive correlation between cyclin D1 and CDK4 or cyclin E and CDK2, the possible roles of these proteins in tumour development and their effects on patient prognosis. The relationships of overexpression of these proteins with various clinicopathological factors were then tested, statistically.

### MATERIALS AND METHODS

### Patients and tumour samples

Ninety cases of primary human oesophageal SCC consecutively obtained at oesophagectomy in the Department of Surgery II, Kochi Medical School between 1982 and 1997 were studied. All patients had received mild chemotherapy with bleomycin (20 mg $m^{-2}$) per day as an oral administration over 5 days, but no radiation therapy prior to surgery. All patients were followed in Kochi Medical School and 23 out of 90 patients received chemotherapy with 5-fluorouracil (5-FU) (1600 mg $m^{-2}$) and CDDP (70 mg $m^{-2}$) as an intravenous infusion over 10 days due to the recurrence. Of the patients, 79 (87.8%) were male and 11 (12.2%) were female. The mean age was 62.2 years (range 41–86 years). In all cases, histological or clinical classification was made using the *Guidelines for Clinical and Pathological Studies on Carcinoma of the Esophagus* established by the Japanese Society for Esophageal Disease (1992). Tumour specimens were fixed in 10% buffered formalin, processed routinely and embedded in paraffin. In each case, all available haematoxylin and eosin-stained sections were reviewed, and a representative block was chosen for further studies.

### Immunohistochemistry with cyclin D1, cyclin E, CDK4 and CDK2 antibodies

Five micrometer-thick sections from archival formalin-fixed paraffin-embedded tissues were placed on poly-L-lysine-coated slides (Sigma Chemical Co., St Louis, MO, USA) for immunohistochemistry (IHC). The expressions of cyclin D1, cyclin E, CDK4 and CDK2 were assessed by immunohistochemical examination using an anti-human cyclin D1 monoclonal antibody (P2D11F11, dilution 1:50; Novocastra, Newcastle, UK), an anti-human cyclin E monoclonal antibody (13A3, dilution 1:50; Novocastra, Newcastle, UK), an anti-human CDK4 polyclonal antibody (dilution 1:500; Pharmingen, San Diego, CA, USA) and an anti-human CDK2 polyclonal antibody (dilution 1:500; Santa Cruz Biotechnology Inc., Santa Cruz, CA, USA) respectively. After blocking of endogenous peroxidase activity, the sections were autoclaved in 10 mM citrate buffer for 12 min at 132°C for antigen retrieval. The deparaffinized sections were pre-treated with normal goat serum for 30 min and incubated with each antibody at 4°C overnight. Immunohistochemical staining for these proteins was then performed using the avidin–biotin complex procedure with a streptavidin–biotin complex peroxidase kit (Histofine SAB-PO Kit; Nichirei Inc., Tokyo, Japan).

The sections were scanned by the two pathologists (MF and YO) simultaneously using a double-headed microscope to identify the areas that were most evenly stained. In each case, 200–500 tumour cells were counted and the percentage of immunoreactivity was determined independently by them. In agreement with a previous study (Ishikawa et al, 1998), immunostaining of cyclin D1 and cyclin E was considered positive if the chromogen was detected in more than 5% of all cancer cells examined. In positive cases, each score was ranked as: 1+, 5–50 positive; or 2+, more than 50% positive.

CDK4 and CDK2 immunoreactivities were observed in both nuclei and cytoplasm of cancer cells and were confined to the basal and parabasal cell layers of non-neoplastic oesophageal epithelium, inflammatory cells, fibroblasts, muscle cells and endothelial cells. This staining was regarded as an internal positive control for immune reaction. Since no study of immunostaining for CDK4 and CDK2 in human oesophageal SCC had previously been performed, we used the same scoring method as their partners, cyclin D1 and cyclin E, described above.

### Statistical analysis

The correlations between cyclin D1 and CDK4 or cyclin E and CDK2 expression as well as the overexpression of these proteins relevant to the various clinicopathological factors were determined using the $\chi^2$ test ($P < 0.05$).

### Association between cyclin D1 and CDK4 or cyclin E and CDK2 overexpression and prognosis

The cumulative survival rates were calculated by the Kaplan–Meier method and the statistical significance of differences was determined using the log-rank test ($P < 0.05$) (with time to death as end-point). A Cox proportional hazards model for risk ratio was also used to assess the simultaneous contributions of cyclins and CDKs immunoreactivities and clinicopathological factors to patient survival.

### RESULTS

### Immunohistochemistry with cyclin D1, cyclin E, CDK4 and CDK2 antibodies

We immunohistochemically examined the expression of cyclin D1, cyclin E, CDK4 and CDK2 in 90 cases of human oesophageal SCC. In total, 28 of 90 (31.1%) tumours exhibited positive staining with cyclin D1-antibody, including five (5.6%) cases of 2+ staining and 23 (25.5%) of 1+ staining. Twenty-seven of the 90 tumours (30.0%), including five (5.6%) cases of 2+ staining and 22 (24.4%) cases of 1+ staining, were positive for cyclin E-antibody. Staining of both cyclin D1 and cyclin E was principally observed in the nucleus of tumour cells. Focal and weak staining of cyclin D1 and cyclin E was observed in normal mucosa adjacent to tumours, but was always restricted to the parabasal cell layer of non-cancerous squamous cell epithelium. Thirty-nine of the 90 tumours (43.3%), including 15 (16.7%) cases of 2+ staining and 24 (26.6%) cases of 1+ staining, and 31 of 90 tumours (34.4%), including five (5.6%) cases of 2+ staining and 26 (28.8%) cases of 1+ staining, were positive in both cytoplasm and nucleus for CDK4 (Figure 1) and CDK2 antibodies respectively. Focal and weak nuclear positive staining of CDK4 (Figure 1) and CDK2 was

© Cancer Research Campaign 1999

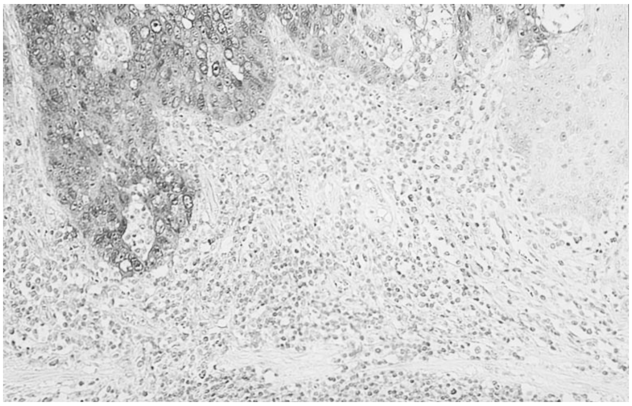

**Figure 1** CDK4 immunoreactivity of oesophageal SCC and non-neoplastic oesophageal epithelium adjacent to tumours. Both cytoplasmic and nuclear immunostainings were observed in cancer cells. Focal and weak nuclear immunopositivity was seen in parabasal layer of non-neoplastic oesophageal epithelium. Reactive follicles and inflammatory infiltrates were also positive

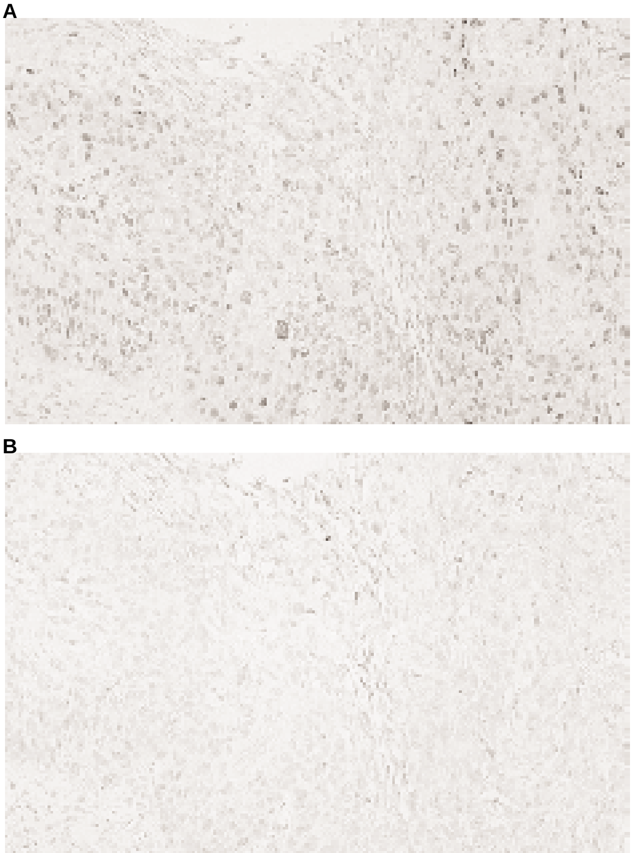

**Figure 2** Cyclin D1 immunopositivity, revealing nuclear predominancy in a case (**A**), but CDK4 (**B**) was negative in the identical case

**Table 1** Correlation of cyclin D1 and CDK4 (a), or cyclin E and CDK2 protein expression (b) in esophageal squamous cell carcinoma

| a | | Cyclin D1 Positive (1+, 2+) | Cyclin D1 Negative (–) | *P*-value |
|---|---|---|---|---|
| | Positive (1+, 2+) | 12 | 27 | |
| CDK4 | | | | >0.1 |
| | Negative (–) | 16 | 35 | |

| b | | Cyclin E Positive (1+,2+) | Cyclin E Negative (–) | *P*-value |
|---|---|---|---|---|
| | Positive (1+,2+) | 7 | 24 | |
| CDK2 | | >0.1 | | |
| | Negative (–) | 20 | 39 | |

**Table 2** Summary of the relationship between the cyclin D1 and CDK4 immunoreactivity and clinicopathological factors in 90 cases of oesophageal SCC

| | Group I (12 cases) | Group II (16 cases) | Group III (62 cases) | Total (90 cases) |
|---|---|---|---|---|
| Age | | | | |
| <60 | 4 | 11 | 22 | 37 |
| 60–70 | 6 | 2 | 25 | 33 |
| >70 | 2 | 3 | 15 | 20 |
| Sex | | | | |
| Male | 10 | 15 | 54 | 79 |
| Female | 2 | 1 | 8 | 11 |
| Histological type | | | | |
| Well | 5 | 2 | 16 | 23 |
| Moderately | 6 | 9 | 40 | 55 |
| Poor | 1 | 5 | 6 | 12 |
| Venous invasion ($P < 0.05$) | | | | |
| (+) | 10 | 5 | 25 | 40 |
| (–) | 2 | 11 | 37 | 50 |
| Lymphatic invasion | | | | |
| (+) | 12 | 11 | 42 | 65 |
| (–) | 0 | 5 | 20 | 25 |
| Lymphnode metastasis | | | | |
| (+) | 7 | 7 | 29 | 43 |
| (–) | 5 | 9 | 33 | 47 |
| Stage | | | | |
| 0,I | 1 | 9 | 23 | 33 |
| II | 3 | 2 | 11 | 16 |
| III | 7 | 3 | 14 | 24 |
| IV | 1 | 2 | 14 | 17 |

Group I: both cyclin D1- and CDK4-positive cases; group II: cyclin D1-positive and CDK4-negative cases; group III: cyclin D1-negative cases.

observed in the basal and parabasal cell layers of normal oesophageal epithelium.

Of cyclin D1- and cyclin E-positive cases, respectively, 12 (42.9%) of 28 and seven (25.9%) of 27 tumours were positive for CDK4 and CDK2, but the remaining 16 (57.1%) and 20 (74.1%) tumours failed to exhibit CDK4 (Figure 2) or CDK2 immunoreactivity.

### Statistical analysis

Statistical analysis was performed using the $\chi^2$ test to determine the correlations of immunoreactivities for cyclin D1 and CDK4, and for cyclin E and CDK2. The results, as shown in Table 1 (a and b), failed to demonstrate a significant relationship among these protein expressions in immunoreactivity.

© Cancer Research Campaign 1999

In addition, we divided the 90 patients into three groups based on cyclin D1 and CDK4 immunoreactivity, i.e. cases positive for both cyclin D1 and CDK4 (group I), cases positive for cyclin D1 but negative for CDK4 (group II) and cases negative for cyclin D1 (group III), and investigated the relationship among these three groups and various clinocopathological factors such as patient age, sex, tumour histological type, vascular and lymphatic invasion, lymph node status and stage. The results, as summarized in Table 2, revealed a significant relationship only between group I and venous invasion ($P < 0.05$). No significant relationship was found between any group and any other clinicopathological parameter. We performed the same type of analysis using the results of staining for cyclin E and CDK2, but no significant relationship was found between any group and any clinicopathological parameter.

No significant relationship in immunoreactivities of any proteins tested between the cases with and without post-operative chemotherapy was detected.

### Association between cyclin D1 and CDK4 or cyclin E and CDK2 overexpression related to patient prognosis

The cumulative survival curves for patients with oesophageal SCC are shown in Figure 3. The outcome of patients with tumours exhibiting simultaneous expression of both cyclin D1 and CDK4 (group I) was significantly poorer than that of patients with cyclin D1-negative tumours (group III) ($P < 0.05$), while no significant difference in outcome was observed between group I and group II. On the other hand, cyclin E and CDK2 overexpressions were unrelated to survivals (data not shown). No prognostic relationship between the patients with and without post-operative chemotherapy was detected.

To determine the effects of predictor variables which were recognized as significant prognostic factors by univariate analysis, including venous invasion, lymphatic invasion, lymph node status, tumour stage and simultaneous immunoreactivity for both cyclin D1 and CDK4, we performed a multivariate analysis using the Cox stepwise proportional hazard model. In this analysis, both cyclin D1 and CDK4 immunoreactivities ($P < 0.01$) and tumour stage ($P < 0.001$) were recognized as independent prognostic factors (Table 3), while other predictors did not retain a significant effect on survival. In addition, group II had poorer survival than group III with a significant hazard ratio of 3.128 (95% confidence interval (CI) = 1.418–6.899, $P = 0.0047$), which was larger than that in group 1 versus group III (hazard ratio (HR) = 2.418, 95% CI = 1.028–5.686, $P = 0.043$) (Table 3).

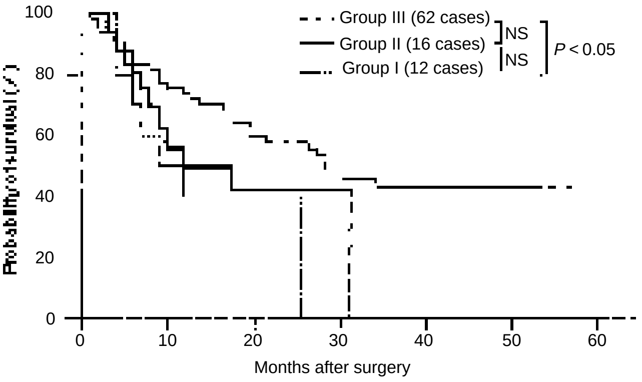

**Figure 3** Cumulative Kaplan–Meier survival curves for patients with oesophageal SCC divided into three groups, i.e. both cyclin D1- and CDK4-positive cases (group I), cyclin D1-positive and CDK4-negative cases (group II), and cyclin D1-negative cases (group III)

**Table 3** Multivariate analysis of prognostic factors by survivals using Cox proportional hazard regression model

| Value | Hazard ratio (95% confidence interval) | *P*-value |
|---|---|---|
| Cyclin D1 and CDK4 | | |
| immunoreactivity | | 0.0088 |
| Group I/Group III | 2.418 (1.028–5.686) | 0.0430 |
| Group II/Group III | 3.128 (1.418–6.889) | 0.0047 |
| Stage | | <0.0001 |
| I/0 | 11.298 (2.510–50.843) | 0.0016 |
| II/0 | 7.957 (2.442–25.928) | 0.0006 |
| III/0 | 10.842 (3.491–33.673) | <0.0001 |
| IV/0 | 19.964 (6.169–64.603) | <0.0001 |

Group I: both cyclin D1- and CDK4-positive cases; group II: cyclin D1-positive and CDK4-negative cases; group III: cyclin D1-negative cases.

### DISCUSSION

We studied the overexpression of cyclin D1, cyclin E, CDK4 and CDK2 proteins in 90 patients with oesophageal SCC, and assessed the relationships in immunoreactivities between cyclin D1 and CDK4, and between cyclin E and CDK2. The relationships between immunopositivity for these proteins and various clinicopathological features and patient prognosis were also statistically examined. Immunohistochemistry using antibodies to cyclin D1, cyclin E, CDK4 and CDK2 made possible precise measurement of the rates of cyclin D1, cyclin E, CDK4 and CDK2 expressions with their patterns of expression in individual tumours, and may be a suitable method for screening of abnormal expression of cyclin D1, cyclin E, CDK4 and CDK2.

The present study revealed that a total of 32.2% (29/90) and 30.0% (27/90) of tumour samples exhibited increased expression of cyclin D1 and cyclin E protein respectively. This expression was observed principally in the nuclei of cancer cells, while totals of 44.4% (40/90) and 35.6% (32/90) of tumour samples exhibited increased expression of CDK4 and CDK2 proteins, respectively, with both cytoplasmic and nuclear immunopositivity. Interestingly, 12 (42.9%) of 28 cyclin D1-positive tumours and seven (25.9%) of 27 cyclin E-positive tumours co-overexpressed their functional partners, CDK4 and CDK2. Furthermore, as expected for these given immunoreactivities, statistical analysis also revealed no significant relationship between cyclin D1 and CDK4 or cyclin E and CDK2 overexpression. A previous study found a significant correlation in mRNA levels between cyclin D1 and CDK4, and between cyclin E and CDK2, in ovarian carcinomas (Masciullo et al, 1997; Marone et al, 1998). Moreover, in human oesophageal cancer cell lines, a significant association between the levels of expression of cyclin D1 protein and in vitro CDK4 enzyme activity has been reported (Doki et al, 1997). On the other hand, there have been a number of recent reports of expression of cyclins and CDKs, including cyclin D1, cyclin E, CDK4 and CDK2, in human malignant tumour cell lines at the DNA, RNA, or protein level (Dirks et al, 1997; Tominaga et al, 1997). The authors of one of these reports commented that variation in expression patterns of these proteins may reflect differences in the biological characteristics of cancer cells and in

© Cancer Research Campaign 1999

the genetic backgrounds acquired during the process of malignant transformation (Tominaga et al, 1997). Our in vivo findings suggest that, although CDKs are catalytic partners of cyclins, in human oesophageal SCC these proteins often exhibit dysregulated overexpression.

We have recently shown that cyclin D1 overexpression is a prognostic marker for human oesophageal SCC (Ishikawa et al, 1997). In the present study, we divided cyclin D1-positive cases into two groups based on immunoreactivity for CDK4, the functional partner of cyclin D1, and evaluated their effects on survival of patients with oesophageal SCC. We found that the patients with the tumours exhibiting simultaneous expression of both cyclin D1 and CDK4 had a poorer prognosis than those with cyclin D1-negative tumours. It has also been reported that abnormal up-regulation of cyclin D1 and CDK4 contributes to malignant progression (Zhang et al, 1997). Our findings also suggest that in human oesophageal SCC, overexpressed cyclin D1 protein promotes the cell cycle progression together with CDK4 in the cyclin D1/CDK4-pRB pathway and contributes to tumour progression. In addition, in a multivariate analysis, patients with cyclin D1-positive and CDK4-negative tumours were found to have a significantly poorer prognosis than those with cyclin D1-negative tumours. Previous studies have shown that overexpression of cyclin D1 protein enhances gene amplification (Asano et al, 1995; Zhou et al, 1996), and that cyclin D1 itself activates oestrogen receptor transcription independent of CDK4 (Neuman et al, 1997; Zwijsen et al, 1997). These studies supported an additional role for cyclin D1 protein independent of the CDK4/cyclin D1-pRB pathway in tumour progression. Therefore, our results also suggest that overexpressed cyclin D1 alone may contribute to tumour progression independent of CDK4 co-expression in oesophageal SCC. In this study, patients underwent mild chemotherapy pre-operatively. We think that cytotoxic agents such as bleomycin might affect the cell cycle. However, all patients were treated identically pre-operatively, so we think chemotherapy alone had little effect on the differences in patients, prognosis. In addition, some patients received post-operative chemotherapy due to recurrence. However, no prognostic relationship between the patients with and without post-operative chemotherapy was detected. Therefore we consider that chemotherapy does not affect our results.

Although the molecular basis for positive immunostaining for cyclin D1, cyclin E, CDK4 and CDK2 remains under investigation, this is the first immunohistochemical study to demonstrate the correlation between these protein expression and clinicopathological factors, and between their immunoreactivities and patients, prognosis in human oesophageal SCC. The present findings suggest that the combinations of cyclin D1 and CDK4, and of cyclin E and CDK2, are immunohistochemically disorder-overexpressed in many cases at the protein level, that simultaneous overexpression of cyclin D1 and CDK4 plays an important role in tumour progression, and that the overexpression of cyclin D1 itself also contributes to tumour progression independent of CDK4 overexpression. More comprehensive studies involving greater numbers of tumours including analyses at the DNA and/or RNA levels will be needed, and examination of CDKIs will also be required to confirm the present findings.

### ACKNOWLEDGEMENTS

The authors are grateful to Dr T Horimi, Kochi Municipal Central Hospital, and Dr T Moriki, Kochi Medical School, for providing materials.

### REFERENCES

Asano K, Sakamoto H, Sasaki H, Ochiya T, Yoshida T, Ohishi Y, Machida T, Kakizoe T, Sugimura T and Terada M (1995) Tumorigenicity and gene amplification potentials of cyclin D1-overexpressing NIH3T3 cells. *Biochem Biophys Res Commun* **217**: 1169–1176

Bartkova J, Lukas J, Guldberg P, Alsner J, Kirkin AF, Zeuthen J and Bartek J (1996) The p16-cyclin D/Cdk4-pRB pathway as a functional unit frequently altered in melanoma pathogenesis. *Cancer Res* **56**: 5475–5483

Dirks PB, Hubbard SL, Murakami M and Rutka JT (1997) Cyclin and cyclin-dependent kinase expression in human astrocytoma cell lines. *J Neuropathol Exp Neurol* **56**: 291–300

Doki Y, Imoto M, Han EKH, Sgambato A and Weinstein IB (1997) Increased expression of the p27kip1 protein in human esophageal cancer cell lines that over-express cyclin D1. *Carcinogenesis* **18**: 1139–1148

Furihata M, Ohtsuki Y, Ogoshi S, Takahashi A, Tamiya T and Ogata T (1993) Prognostic significance of human papillomavirus genomes (type-16, -18) and aberrant expression of p53 protein in human esophageal cancer. *Int J Cancer* **54**: 226–230

Furihata M, Ishikawa T, Inoue A, Yoshikawa C, Sonobe H, Ohtsuki Y, Araki K and Ogoshi S (1996) Determination of the prognostic significance of unscheduled cyclin A overexpression in patients with esophageal squamous cell carcinoma. *Clin Cancer Res* **2**: 1781–1785

Igaki H, Sasaki H, Kishi T, Sakamoto H, Tachimori Y, Kato H, Watanabe H, Sugimura T and Terada M (1994) Highly frequent homozygous deletion of the p16 gene in esophageal cancer cell lines. *Biochem Biophys Res Commun* **203**: 1090–1095

Ishikawa T, Furihata M, Ohtsuki Y, Ono H, Inoue A and Ogoshi S (1997) Aberrant expression of p53 and retinoblastoma gene products in human esophageal squamous cell carcinoma. *Int J Oncol* **11**: 1109–1114

Ishikawa T, Furihata M, Ohtsuki Y, Murakami H, Inoue A and Ogoshi S (1998) Cyclin D1 overexpression related to retinoblastoma protein expression as a prognostic marker in human esophageal squamous cell carcinoma. *Br J Cancer* **77**: 92–97

Jiang W, Zhang YJ, Kahn SM, Hollstein MC, Santella RM, Lu SH, Harris CC, Montesano R and Weinstein IB (1993) Altered expression of the cyclin D1 and retinoblastoma genes in human esophageal cancer. *Proc Natl Acad Sci USA* **90**: 9026–9030

Khatib ZA, Matsushime H, Valentine M, Shapiro DN, Sherr CJ and Look AT (1993) Co-amplification of the CDK4 gene with MDM2 and GLI in human sarcomas. *Cancer Res* **53**: 5535–5541

Lee MH, Reynisdottir I and Massague J (1995) Cloning of p57KIP2, a cyclin-dependent kinase inhibitor with unique domain structure and tissue distribution. *Genes Dev* **9**: 639–649

Marone M, Scambia G, Giannitelli C, Ferrandina G, Masciullo V, Bellacosa A, Benedetti Panici P and Mancuso S (1998) Analysis of cyclin E and cdk2 in ovarian cancer: gene amplification and RNA overexpression. *Int J Cancer* **75**: 34–39

Masciullo V, Scambia G, Marone M, Giannitelli C, Ferrandina G, Bellacosa A, Benedetti Panici P and Mancuso S (1997) Altered expression of cyclin D1 and CDK4 genes in ovarian carcinomas. *Int J Cancer* **74**: 390–395

Motokura T and Arnold A (1993) Cyclin D1 and oncogenesis. *Curr Opin Genet Dev* **3**: 5–10

Naitoh H, Shibata J, Kawaguchi A, Kodama M and Hattori T (1995) Overexpression and localization of cyclin D1 mRNA and antigen in esophageal cancer. *Am J Pathol* **146**: 1161–1169

Nakamura T, Sanokawa R, Sasaki YF, Ayusawa D, Oishi M and Mori N (1995) Cyclin I: A new cyclin encoded by a gene isolated from human brain. *Exp Cell Res* **221**: 534–542

Neuman E, Ladha MH, Lin N, Upton TM, Miller SJ, Direnzo J, Pestell RG, Hinds PW, Dowdy SF, Brown M and Ewen ME (1997) Cyclin D1 stimulation of estrogen receptor transcriptional activity independent of cdk4. *Mol Cell Biol* **17**: 5538–5347

Sherr CJ (1993) Mammalian G1 cyclins. *Cell* **73**: 1059–1065

Sherr CJ (1994) G1 phase progression: cyclins on cue. *Cell* **79**: 551–555

Sherr CJ (1996) Cancer cell cycles. *Science* **274**: 1672–1677

Sheyn I, Noffsinger AE and Heffelfinger S (1997) Amplification and expression of the cyclin D1 gene in anal and esophageal carcinomas. *Hum Pathol* **28**: 270–276

Tominaga O, Nita ME, Nagawa H, Fujii S, Tsuruo T and Muto T (1997) Expressions of cell cycle regulators in human colorectal cancer cell lines. *Jpn J Cancer Res* **88**: 855–860

Tsuruta H, Sakamoto H, Onda M and Terada M (1993) Amplification and overexpression of EXP1 and EXP2/cyclin D1 genes in human esophageal carcinomas. *Biochem Biophys Res Commun* **196**: 1529–1536

© Cancer Research Campaign 1999

Wang LD, Shi ST, Zhou Q, Goldstein S, Hong JY, Shao P, Qiu SL and Yang CS (1994) Changes in p53 and cyclin D1 protein levels and cell proliferation in different stages of human esophageal and gastric-cardia carcinogenesis. *Int J Cancer* **59**: 514–519
Weinberg RA (1995) The retinoblastoma protein and cell cycle control. *Cell* **81**: 323–330
Zhang T, Nanney LB, Luongo C, Lamps L, Heppner KJ, DuBois RN and Beauchamp RD (1997) Concurrent overexpression of cyclin D1 and cyclin-dependent kinase 4 (CDK4) in intestinal adenomas from multiple intestinal neoplasia (Min) mice and human familial adenomatous polyposis patients. *Cancer Res* **57**: 169–175
Zhou P, Jiang W, Weghorst CM and Weinstein IB (1996) Overexpression of cyclin D1 enhances gene amplification. *Cancer Res* **56**: 36–39
Zwijsen RM, Wientjens E, Klompmaker R, van der Sman D, Bernards R and Michalides RJ (1997) CDK-independent activation of estrogen receptor by cyclin D1. *Cell* **88**: 405–415

*© Cancer Research Campaign 1999*
